# WFDC Protein: A Promising Diagnosis Biomarker of Ovarian Cancer

**DOI:** 10.7150/jca.57880

**Published:** 2021-07-06

**Authors:** Chen Zhang, Haoyue Hu, Xiaoyan Wang, Yajuan Zhu, Ming Jiang

**Affiliations:** 1 State Key Laboratory of Biotherapy, Cancer Center, West China Hospital, Sichuan University, Chengdu, Sichuan Province, China; 2 Lung Cancer Center, Cancer Center, State Key Laboratory of Biotherapy, West China Hospital of Sichuan University, Chengdu, Sichuan, 610041, China; 3 West China Hospital, Sichuan University, Chengdu, People's Republic of China

## Abstract

An initial diagnosis of cancer is usually based on symptoms, abnormal physical examination and imaging tests. Ovarian cancer is difficult to be diagnosed timely due to the nonspecific symptoms, thus resulting in the high-risk mortality. Despite of the various diagnostic methods, there is still no reliable diagnostic test. Clinically, carbohydrate antigen 125(CA125) is widely recognized as a diagnosis biomarker of ovary cancer. However, CA125 is not sensitive to detect the ovary cancer at the early stage. It is essential to explore other potential biomarkers. Human epididymis protein 4 (HE4) in the whey/four-disulfide core (WFDC) proteins family shows satisfactory sensitivity in the early diagnosis of ovary cancer. In this present review, we summarized the important effects of WFDC family proteins on the proliferation, apoptosis and migration of ovary cancer and intended to provide more evidence to explore the possibility of WFDC protein as a diagnosis biomarker.

## Introduction

Although ovarian cancer accounts for only a third of gynecologic cancers, it results in 55% of deaths from gynecologic malignancies and 6% of all cancer deaths in women. The 50~70-year-old group is a group with the highest incidence of disease 1. Ovarian cancer with high mortality rate among women has attracted much attention. Although it is not as common as breast cancer, delayed diagnosis may result in distant metastasis and high lethality. Despite advances are being made, ovarian cancer remains the most fatal female gynecologic cancer 2. Early detection of the disease can significantly improve the cure rate of ovarian cancer. Therefore, the use of biomarkers is extremely important in the diagnosis of ovarian cancer.

In 1980, 'four-disulfide core' (FDC) structure and overall folding patterns have been initially found between snake venom postsynaptic neurotoxin and wheat germ agglutinin domain. This structure is composed of four similar disulfide bonds with different size ring 3. Then, Hennighausen *et al.* confirmed that whey acidic protein was the main protein in the milk of rats and mice. The whey-acidic-protein motif in this protein is the prototypic member of a subfamily of the whey/four-disulfide core proteins (WFDC) 4. The gene encoding proteins of this family are located on human chromosome 20q13. And the proteins are defined by the possession of one or more 40-50 amino acid domains, including eight conserved cysteine residues linked by four characteristic intramolecular disulfide bonds 5. The proteins of WFDC family have multiple active functions, such as anti-HIV, anti-microbial, immune and cell migration activities 6.

With the emergence of selective molecular targeted therapies, biomarkers play an increasingly crucial role in the clinical management of cancer patients 7. The monitoring of biomarkers can predict the existence, incidence and prognosis of tumors, providing help to clinical treatment and medication guidance. Among various discovered biomarkers, carbohydrate antigen 125 (CA125) is the most commonly used blood-based biomarker for ovarian cancer diagnostic. However, CA125 is elevated in some case of certain common benign diseases, such as endometriosis, follicular cysts, pregnancy and cystadenoma, indicating that CA125 lacks the specificity to predict ovarian cancer 8. In addition, CA125 detected in the serum of early ovarian cancer patients has quite lower sensitivity than in the advanced stage patients 9. Thence, it is essential to discover biomarkers that have both high sensitivity and early identification in ovarian cancer. Later, human epididymis protein 4(HE4) protein was found to be highly expressed in ovarian cancer 10. As a promising biomarker for epithelial ovarian cancer, it shows better specificity and sensitivity in detection 11. Therefore, it has received widespread attention as a cancer biomarker in recent years. Additionally, the simultaneous measurement of indicators HE4 and CA125 in serum improves the accuracy of ovarian cancer diagnosis and provides the distinction between ovarian tumors and endometriotic cysts 12. Elevated evidence suggests that WFDC family proteins with whey-acidic-protein (WAP) domain have potential on becoming ranks of biomarker for their diverse functions. We intend to summarize the role of WFDC family with WAP domain as biochemical markers of ovarian cancer.

## WFDC family and ovarian cancer

**Figure 1:**
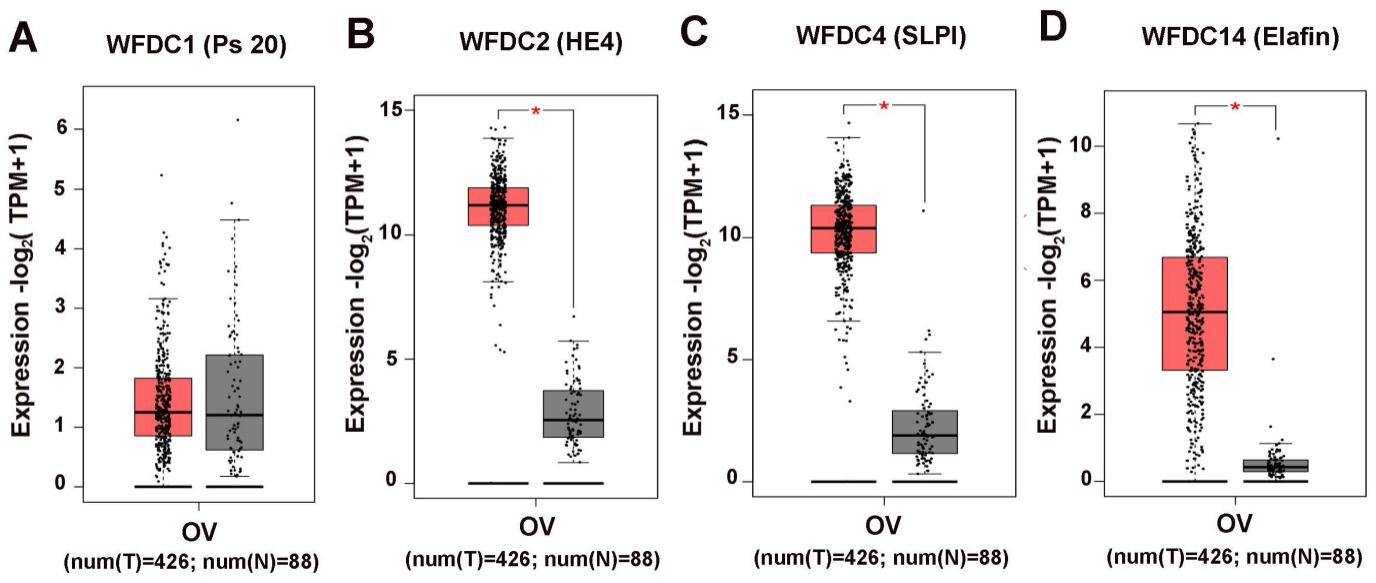
**The expression levels of WFDC proteins in ovarian cancer.** (A) WFDC1 (B) WFDC2 (C) WFDC4 (D)WFDC14. The red represents the tumor tissue, the gray represents the adjacent tissues , each point represents the expression of the sample (*P<0.05).

**Figure 2:**
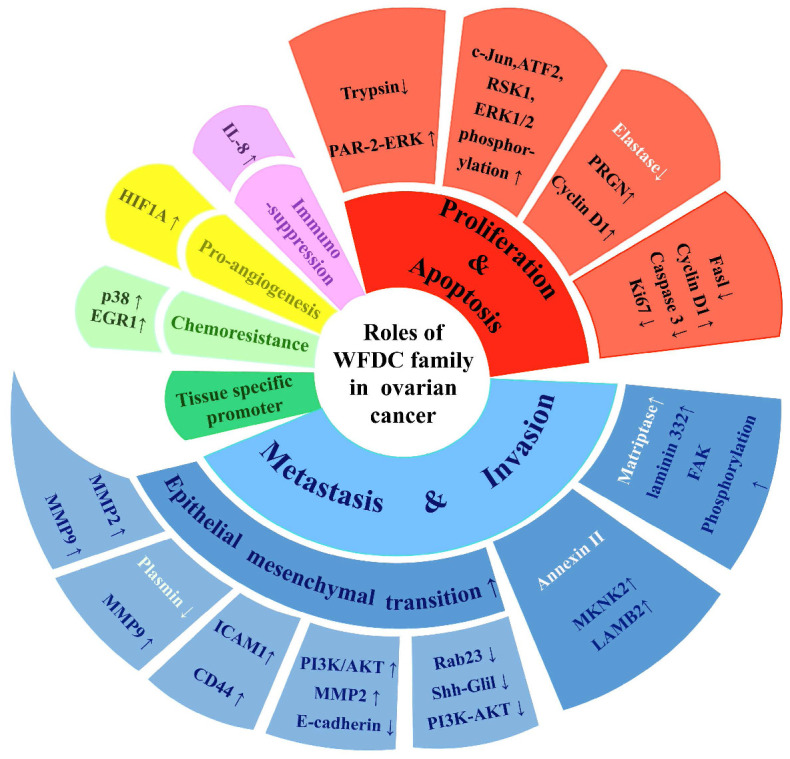
** The multiple functions of WFDC family proteins in ovarian cancer.** WFDC family proteins have various roles in ovarian cancer, including cell proliferation and apoptosis, migration and invasion, immunosuppression, pro-angiogenesis, chemoresistance, as well as treatment potential as tissue-specific promoters. The white part of the font indicates that the mode of action is not directly through the protease inhibitor activity.

The attention of researches on cancer has gradually focused on genes coding WFDC family proteins. Most notably, the genes coding for WAP four disulfide core domain protein 14 (WFDC14, also named elafin), WAP four disulfide core domain protein 4 (WFDC4 also named SLPI), WAP four disulphide core domain protein 1 (WFDC1, also named PS20) and WAP four disulphide core domain protein 2 (WFDC2, also named HE4), have been studied on ovarian cancer. In the past ten years, SLPI and HE4 were the most studied molecules of the WFDC family in pathogenesis and development of ovarian cancer 10, 13. In 2010, Elafin was linked with ovarian cancer 14. Recently, reports on other proteins of WFDC family, such as Elafin and ps20, are gradually increasing 15-17. These family proteins with WFDC-type protease inhibition activity are encoded by gene localized in chromosome 20q12-q13. It has the frequent amplification region in ovarian cancer, and the WFDC protein family genes located in this chromosome are amplified in several cancers, such as ovarian, breast, colon and prostate cancer. The analyses of data from the Cancer Genome Atlas Program (TCGA) show that HE4, SLPI and elafin all showed significant difference in ovary cancer, while the ps20 proteins expression had no difference (**Figure 1**). Among these, HE4 has been repeatedly confirmed to have significant expression changes in ovarian cancer tumor. Related basic experiments have proved that HE4 has an impact on tumor progression, such as proliferation, apoptosis invasion and migration 18, 19. Of note, metastasis and invasion of early-stage ovarian cancer is a major factor responsible for its high mortality and poor prognosis 20. Elafin, SLPI and ps20 have limited research reports on ovarian cancer. However, these proteins may affect various crucial cancer behaviors through multiple functions (**Figure 2**), the same as HE4, indicating the crucial role of WFDC family proteins as biomarkers.

## HE4 in ovarian cancer

HE4 is currently most studied in ovarian cancer. It has been currently evaluated for diagnosing ovarian malignant tumor as a novel biomarker 21. In the epithelium tissues of respiratory and reproductive organs, HE4 is weakly expressed. On contrast, HE4 is overexpressed in ovarian tumor, especially in endometrioid ovarian cancer 10. In addition, the overexpression of HE4 can enhance proliferation, apoptosis, invasion and metastasis of ovarian cancer 19, 22. A previous study has revealed that silencing of HE4 can lead to impaired capability of human serous ovarian carcinoma cell line SKOV3, such as proliferation, cell cycle, early apoptosis ability of invasion and migration. Meanwhile, it has been confirmed that decrease of these abilities were related to phosphorylated protein of the ERK pathway, JAK-STAT pathway and matrix metalloproteinases 23, 24. HE4 as a serine protease inhibitor can also augment the activity of trypsin, thereby enhancing proteinase activated receptor 2 (PAR2)-mediated cell proliferation by ERK pathway 25. J.R. Ribeiro et al found that HE4 contributes to the collateral resistance to cisplatin and paclitaxel in HE4-overexpression cell lines. The MAPK-regulated response gene involved in promoting metastasis and apoptosis, such as p38 and EGR1, increased 26.

It is interesting that HE4 has an essential effect on tumorigenesis and development without directly exerting its serine protease inhibitory function 27, 28. In vitro, matriptase activity was enhanced by recombinant HE4 in a dose-dependent manner. It indicated that HE4 can enhance matriptase enzymatic activity of matriptase and, possibly working in concert with upregulation of laminin-332 by HE4 to promote laminin-332 functions, thus affecting migration, invasion, or adhesion. Meanwhile, this study confirmed that HE4 promotes the activation of FAK signaling pathway to stimulate cell adhesion to the extracellular matrix 29, 30. Furthermore, there have been several studies reported about the interaction between HE4 and Annexin II (ANXA2) protein by a specific binding model in endometrial carcinoma 31, 32. As a calcium-dependent phospholipid binding protein annexin ANXA2 II may help HE4 translocate into the nucleus, where it functioned as a transcription factor and increased the expression of MAPK or FOCAL signaling molecules, MAPK interacting serine/threonine kinase 2 (MKNK2) and laminin subunit beta 2 (LAMB2), to promote ovarian cancer cell invasion and metastasis 18. And HE4 not only regulated the expression of Fasl, cyclin D1, caspase 3 and Ki67 to influence tumor apoptosis and proliferation, but also altered metastasis gene expression such as intercellular adhesion molecule 1 (ICAM-1), CD44, matrix metalloproteinase-2 (MMP2) and matrix metalloproteinase-9 (MMP9) to influence tumor metastasis 22, 24, 33. The epithelial-mesenchymal transition (EMT) process in the progression of cell migration and invasion, as the key point of ovarian metastasis, was influenced by HE4, which promotes the polarization of epithelial cells and imparts mesenchymal cell characteristics. Later, it was proposed that HE4 activated PI3K/AKT signaling pathway and increased the MMP2 expression to influence EMT 34. The knockdown of HE4 reduced the level of MMP2 and MMP9, further limiting EMT process 24. In addition, HE4 can upregulate Rab23 protein expression, which also involved in EMT process of ovarian cancer cells by acting Shh-Gli1 and PI3K-AKT signaling pathways 27, 35. The role of HE4 in affecting metastasis as an expression regulator of EMT is still required further evaluation.

Meanwhile, HE4 contributes to occurrence of inflammation in tumor environment. Recently, it was the first time to confirm that HE4 suppresses STAT3 to upregulate IL8 and HIF1A gene expression. This progress affects microenvironment and angiogenesis of ovarian cancer. Expression of HE4 was associated with reduced CD8^+^ T cells in tissue of epithelial ovarian cancer patients 36. Collectively, considering the important role of HE4 in proliferation, metastasis, chemoresistance and immune suppression, HE4 can plausibly be targeted for therapeutic benefit and immunomodulatory effects.

## SLPI in ovarian cancer

SLPI is reported to overexpress in ovarian cancer while it has low expression levels in normal organs 37, 38. And upregulation in human ovarian cancer cells was detected upon exposure to paclitaxel, resulting in chemoresistance in part through ERK1/2 activation 39. Similarly, Elafin can also affect the EMT process, which does not exclusively depend on protease inhibitor activity. The overexpression of SLPI independently on the protease inhibition function is associated with increased proliferation and metastasis of ovarian cancer. SLPI can protect progranulin (PGRN) from proteolysis by binding to the inter-granulin linker areas to block the accessibility to proteases, or inhibiting the converting protease elastase directly 40. The previous study found that SLPI can protect PRGN from serine protease-mediated degradation. The protective effect of SLPI on PRGN was independent of its protease inhibitory activity and increased cyclin D1 expression proliferation of ovarian cancer 41. The indirect blocking role is also confirmed by other studies. The protease inhibition-null mutant (PI-/SLPI) can partner with PRGN and provide equal or greater protection for PRGN from elastase 42. It also has been demonstrated that SLPI had a regulatory direct or indirect effect on MMP quantity through transcriptional upregulation in the nucleus of ovarian cancer cell 43, even though it was lack of a nuclear localization signal. Meanwhile, coupling with its extracellular interaction with plasmin, SLPI inhibited plasmin activity to regulate MMP-9 activation and release. MMP9 is an essential factor of selectively modulating the tumor microenvironment and promoting tumor cell development as an inducer of EMT 44, bringing to light that modulation of activity of SLPI may be therapeutically relevant in ovarian cancer, especially influencing metastasis.

Applying to SLPI as tissue-specific promoter is a potentially useful method for ovarian cancer and allows targeted options for better gene therapy of ovarian cancer. In the ovarian tumor, gene expression regulated by the SLPI promoter was shown to be higher than in normal tissues such as the liver, lung, heart, kidney, and so on. Under the expression control of the SLPI promoter, HSVtk/GCV-mediated cell killing for ovarian cancer considerably improved survival in mice model to a comparable extent as the CMV promoter 45.

## Elafin in ovarian cancer

The first report connecting Elafin to ovarian cancer was presented on 2010. Elafin expression correlated with poor overall survival in late-stage, high-grade serous ovarian carcinomas. Importantly, its expression can be transcriptionally upregulated by immune cytokines via the activation of NF-κB pathway in primary tumors 14. In high-grade serous ovarian cancer, Elafin overexpressed and was secreted by advanced ovarian cancer, leading a proliferative impact by phosphorylation of c-Jun (S63), ATF2 (S90), ribosomal protein S6 kinase (RSK1) and ERK1/2 through the MEK-ERK pathways 46. However, another study discovered that Elafin-positive cells were predictors of poor disease-specific survival only in stage I/II ovarian cancer patients. Compared to the normal fallopian tube, Elafin also downregulated in 33% of ovarian cystadenomas, 43% of borderline ovarian tumors, and 86% of invasive ovarian carcinomas 15. Human epithelial ovarian cells were less sensitive to genotoxic drugs such as cisplatin, carboplatin, cyclophosphamide, and 5-fluorouracil after treatment with Elafin. This effect of cisplatin-resistance caused by Elafin suppressed the cisplatin-induced apoptosis and caspases activation 47. Despite the fact that Elafin individually is not a highly specific and sensitive diagnostic biomarker for epithelial ovarian cancer now, the monitoring of serum Elafin in combination of CA125 and HE4 may also provide clinically applicable information.

## PS20 in ovarian cancer

Prostate stromal 20 (ps20) is increasingly recognized as an important regulator of tumor growth, mostly in progressive prostate cancer 48, 49. Previous researches have mostly explored how ps20 might impact prostate cancer behaviors 49, 50. The latest reports highlighted that sorbin and SH3 domain containing 2 (SORBS2) binds the 3' untranslated regions (UTRs) of ps20 to enhance the stability of these gene transcripts, which suppresses the invasiveness of ovarian cancer 17. In addition, ps20 expression was shown to be substantially lower in metastatic ovarian cancer sites as compared to original sites by examining the Oncomine database. Furthermore, there was positive correlation between ps20 expression with overall survival of ovarian cancer patients 17. It has been shown that ps20 may be intimately linked to disease migration WFDC protein as a biomarker in the detection of early-stage ovarian cancer seems promising, according to further in-depth mechanistic study.

## WFDC protein can be recognized as promising biomarker of diagnosis and treatment in ovarian cancer

In comparison to other cancers associated with women, 77% of them are diagnosed as endometrial cancers stage I, 55% of breast cancers and 83% of cervical cancers respectively. While only 23% of ovarian malignancies are detected at an early stage 51. Therefore, there are no clear indicators that can be utilized in clinic. The early diagnosis of ovarian cancer is continuously being researched. Successful screening of ovarian cancer is necessary. The CA125 was firstly described in the early 1980's. The identification of CA125 as a circulating antigen paved the way for ovarian carcinoma biomarker research 52. Then, several various tumor biomarkers have been evaluated via laboratory tests (**Table 1**).

CA125 is originally used to monitor the chemotherapy response of ovarian cancer and detect the relapse after surgery 53, which is a peptide epitope of 3-5 million Da mucin (MUC16) 54. MUC16, commonly known as CA125, is not only a biomarker for ovarian cancer, but also evolves in tumor progression and metastasis 55. Due to their aberrant overexpression and functional involvement, MUC16 and its ligands have emerged as prospective targets for therapeutic intervention utilizing monoclonal antibodies and immunotherapy 56, 57. However, cleavage of extracellular MUC16 domain, an inadequate understanding structural variety of the MUC16 domains and uncharacterized associated signaling pathway under illness circumstances all provide obstacles to the therapeutic development of MUC16 or CA125 57. Recently, levels of CA125 have recently been universally acknowledged as increasing in other several benign conditions. CA125 elevates in 80% of patients with epithelial ovarian cancer at initial diagnosis and correlates strongly with response to therapy, while it has extremely limited sensitivity in identifying patients in early-stage ovarian cancer 58.

In a randomized controlled trial, annual multimodal screening with serum CA125 and transvaginal ultrasound sonography as a second-line diagnostic demonstrated minimal effectiveness 59. A recent research has suggested that discovering biomarkers in urine can provide a non-invasive technique for detecting ovarian cancer, and allow the frequent testing of women with high-risk. In contrast, CA125, a typical serum tumor biomarker in ovarian cancer, showed no significance in the urine data 60.

Secretory HE4 showed significant difference as urinary biomarker in the differential diagnosis between ovarian cancer and benign tumors, followed by creatinine, carcinoembryonic antigen, vascular cell adhesion molecule (VCAM) and transthyretin (TTR) 60. The HE4 expression levels in serum and urine of ovarian cancer patients was significantly upregulated compared with normal or benign groups 24. HE4 as a biomarker in early-stage cancer was not overexpressed in benign ovarian disease, normal ovarian tissue or tumors with low malignant potential 61. Meta-analysis from multiple studies systematically evaluated the potential of urine HE4 as a non-invasive biomarker for the diagnosis of ovarian cancer 62. In this meta-analysis, four Asian studies had explicitly evaluated the diagnostic value of urine HE4 and serum HE4, demonstrating that urine HE4 had a higher pooled sensitivity (0.82 vs. 0.74) and a higher pooled specificity (0.93 vs 0.88) compared to serum HE4. The area under the curve (AUC) of summary receiver operating characteristic (SROC) for urine HE4 and serum HE4 were 0.92 and 0.95, respectively, which indicated that urine HE4 may be superior to serum HE4 in screening ovarian cancer. Earlier research indicated that compared to concentration of serum HE4, urinary concentrations of HE4 elevated in patients with early and advanced ovarian cancer, as well as in patients with serous ovarian cancer 63, which uncovered that urinary HE4 may be more sensitive than serum HE4 analysis in certain cases of ovarian cancer. The protein profile in urine is less complicated than that in blood, thus overall clinical performance is enhanced by the measurement of urinary proteins. Meanwhile, certain proteins may be more stable in urine than in blood. Moreover, urinary tests would be more convenient than invasive blood tests. HE4, with a molecular weight of 25 kDa, is below the glomerular filtration cutoff 64. WFDC family including HE4 is composed of small secretory molecular protein, so it may be worth expanding areas for urine biomarker to detect early-stage ovarian cancer. Simultaneously, it has potential to create novel methods of ovarian cancer monitoring, such as monitoring through urine while enhancing specificity and sensitivity.

Diagnostic comparisons of potential single biomarker, including HE4, CA125, carcinoembryonic antigen (CEA), carbohydrate antigen 19-9 (CA19-9), transthyretin (TTR) and apolipoprotein A-1 (ApoA-1), also indicate HE4 has important roles in diagnosing 65-67, particularly in early-stage **(Table 1)**. The serum CA125 and computed tomography (CT) imaging are currently used for evaluating primary therapy response evaluation in epithelial ovarian cancer 68, while the postoperative CA125 level is an unreliable indicator for residual tumor. Instead, postoperative HE4 is statistically significantly associated to residual tumor and first line chemotherapy after primary debulking surgery and interval debulking surgery in patients with advanced epidermal ovarian carcinoma 69. Several clinical investigations assess the sensitivity and specificity of HE4 and CA125 indicators. Unlike CA125, serum levels of HE4 are not elevated in common benign conditions, such as pregnancy and endometriosis. CA125 has higher sensitivity than HE4 in both premenopausal and postmenopausal women, while HE4 has the highest specificity in both menstrual states 70-72** (Table 1)**. Several clinical trials have noted that HE4 levels had equal sensitivity but higher specificity in detecting ovarian cancer when compared to CA125 levels 72, 73. SLPI, which belongs to the same family as HE4, has been shown to be an excellent predictor of epidermal ovarian cancer 74. Currently, HE4 protein is still the most researched protein of WFDC family which is used for ovarian cancer biomarker in clinical trials. Efficacy of other WFDC family proteins needs further researched by clinical trial.

Advances in biomarker discovery have led to several FDA-approved tests superior to CA125 in preoperative evaluation of women with a pelvic mass, including ROMA, Overa and OVA1 test (**Table 2**). It is an initial enthusiasm to estimate the possibility of malignancy as a triage to ovarian masses and diagnostic aid, although they are not ideal diagnostic tests for early-stage detection. It presents a trend of variations that exploring biomarkers alternatives to CA125 alone and complements diagnostic performance in early-stage. A new insight of combination of markers to detect the occurrence and development of early ovarian cancer may improve the sensitivity and accuracy of detection (**Table 2**). Multiple biomarker panels, including HE4 protein, are being studied in depth 75, 76. Combined measurements HE4 and CA125 can also be useful in differential diagnosis of different pathological type of ovarian cancer, especially diagnosis between ovarian endometrioma and epithelial ovarian cancer 77. Studies using the combined biomarkers in ovarian cancer patients are warranted. And it has great potential in exploring new biomarkers complemented to the early diagnostic.

## Summary and Prospects

WFDC family proteins are involved in several aspects of the occurrence and development of ovarian cancer, including proliferation, apoptosis, invasion and metastasis. HE4 Ovarian Cancer Monitoring Trial set up to measure the HE4 protein level in blood for the diagnosis. Basic researches and clinical trials are expected to investigate the efficacy of other molecules of WFDC family compounds as biomarker in monitoring, diagnosis and treatment of ovarian cancer, especially in early-stage. We anticipate that further studies on the sensitivity and specificity of molecules other than HE4 in clinical trials will be undertaken to aid in the diagnosis, treatment and prognostic monitoring of early-stage ovarian cancer.

## Author Contributions

Chen Zhang wrote the original draft. Haoyue Hu revised the manuscript. Xiaoyan Wang summarized the literature. Yajuan Zhu provided an overall idea. Ming Jiang modified the framework and re-designed pictures and tables.

## Figures and Tables

**Table 1:** Comparison of diagnostic performance of multiple biomarker alone in clinical analysis.

Biomarker	Subject	Grouping	Physiological Condition	SN(%)	SP(%)	PPV(%)	NPV(%)	ROC	Cutoff	Reference
CA125	419	EOC:114/27.2%	PreM	92.3	59.4	_	_	_	35 U/ml	78
			PostM	94.3	82.3	_	_	_		
	373	Malignancy:55/14.7%	PreM	85.7	55.7	_	_	_	35 U/ml	79
			PostM	88.1	85.0	_	_	_		
	458	Malignancy:196/42.8%		62.8	70.6	_	_	0.717	35 U/ml	80
	263	OC:118/44.9%	stage I & II	51.6	51.2	_	_	_	35 U/ml	81
			stage III & IV	94.3	84.3	_	_	_		
HE4	419	EOC:114/27.2%	PreM	84.6	94.2	_	_	_	70 pM/L	78
			PostM	78.2	99.0	_	_	_	140 pM/L	
	373	Malignancy:55/14.7%	PreM	57.1	100.0	_	_	_	140 pM/L	79
			PostM	71.2	10.0	_	_	_		
	458	Malignancy:196/42.8%	_	63.8	96.6	_	_	0.967	140 pM/L	80
CEA	458	Malignancy:196/42.8%	_	38.8	88.6	_	_	0.777	5 ng/ml	80
	266	OC:19/7.1%	_	11.1		11.2	95.0	_	4.6 ng/ml	82
CA19-9	458	Malignancy:196/42.8%	_	35.7	79.0	_	_	0.624	27 U/ml	80
	266	OC:19/7.1%	_	16.7	_	15.8	95.4	_	43 kIU/L	82
TTR	256	OC:126/49.2%	stage I & II	78.6	_	68.8	_	0.912	_	83
			stage III & IV	82.9	_	74.3	_	0.932	_	
	263	OC:118/44.9%	stage I & II	61.2	59.2	_	_	_	100 ng/ml	81
			stage III & IV	74.9	68.2	_	_	_		
ApoA-1	263	OC:118/44.9%	stage I & II	51.3	54.6	_	_	_	500 ng/ml	81
			stage III & IV	58.4	55.1	_	_	_		

CEA: carcinoembryonic antigen; CA19-9: Carbohydrate antigen 19-9; TTR: Transthyretin; ApoA-1: Apolipoprotein A-1. EOC: Epithelial ovarian cancer; OC: Ovarian cancer; SN: Sensitivity; SP: Specificity; PPV: Positive predictive value; NPV: Negative predictive value; ROC: Receiver operator characteristic.

**Table 2:** Comparison of diagnostic performance of multimarker test in clinical analysis.

Multimarker Test	Subject	Grouping	Physiological Condition	SN(%)	SP(%)	PPV	NPV	Cutoff	Reference
CA125+HE4+ CEA+VCAM-1	1097	OC:168/15.3%	stage I & II	86	98%	_	_	_	84
			stage III & IV	95		_	_	_	
CA125+HE4+E-CAD+IL-6	171	OC:125/73.1%	_	84.2	95.7	88.9	93.6	_	76
ROMA+	419	EOC:114/27.2%	PreM	84.6	81.2	_	_	preM 7.4%; postM 25.3%	78
			PostM	93.1	84.4	_	_		
	472	EOC:48/10.2%	PreM	100	74.2	17.8	97.4	preM 13.1%; postM 27.7%	85
			PostM	92.3	76	56.1	100		
	373	Malignancy:55/14.7%	PreM	72.5	79.1	_	_	preMindex11.4; postM index 29.9	79
			PostM	87.3	91.7	_	_		
	458	Malignancy:196/42.8%	_	81.1	84	_	_	_	80
OVA1++	493	Malignancy:92/18.7%%	_	94.2	53.5	31.4	96.8	_	86
Overa*	493	Malignancy:92/18.7%%	_	91.3	69.1	40.4	97.2	_	86
	218	EOC:64/29.4%	PreM	78.3	72.3	58.1	87.2	_	87
			Post M	90.1	62.3	68.8	87.3	_	

+, Roma Algorithm; *, CA125, HE4, transferrin (TRF), apolipoprotein A-1 (ApoA-1) and follicle-stimulating hormone (FSH). ++, CA 125, transferrin (TRF), transthyretin (TTR) , apolipoprotein A1 (ApoA1), and β 2-microglobulin (B2M) ; EOC: Epithelial ovarian cancer; OC: Ovarian cancer; SN: Sensitivity; SP: Specificity; PPV: Positive predictive value; NPV: Negative predictive value.

## Abbreviations

CA125: carbohydrate antigen 125
HE4: human epididymis protein 4
WFDC: whey/four-disulfide core
WFDC1: WAP four disulfide core domain protein 1
WFDC2: WAP four disulfide core domain protein2
WFDC4: WAP four disulfide core domain protein 4
WFDC14: WAP four disulfide core domain protein 14
PAR2: proteinase activated receptor 2
ANXA2: annexin II
EMT: epithelial mesenchymal transition
MKNK2: MAPK interacting serine/threonine kinase 2
Ps20: prostate stromal 20
LAMB2: laminin subunit beta 2
ICAM-1: intercellular adhesion molecule 1
MMP2: matrix metalloproteinase 2
MMP9: matrix metalloproteinase 9
PRGN: protect progranulin
RSK1: ribosomal S6 kinase 1
SORBS2: sorbin and SH3 domain containing 2
UTRs: untranslated regions
VCAM: vascular cell adhesion molecule
TTR: transthyretin
AUC: area under the curve
SROC: summary receiver operating characteristic
CA19-9: carbohydrate antigen 19-9
ApoA-1: apolipoprotein A-1
